# A neurologist’s perspective on serum neurofilament light in the memory clinic: a prospective implementation study

**DOI:** 10.1186/s13195-021-00841-4

**Published:** 2021-05-18

**Authors:** E. A. J. Willemse, P. Scheltens, C. E. Teunissen, E. G. B. Vijverberg

**Affiliations:** 1 Neurochemistry Laboratory, Department of Clinical Chemistry, Amsterdam Neuroscience, Amsterdam University Medical Center, Vrije Universiteit, De Boelelaan, 1117 Amsterdam, The Netherlands; 2 Alzheimer Center, Department of Neurology, Amsterdam Neuroscience, Amsterdam University Medical Center, Vrije Universiteit, De Boelelaan, 1117 Amsterdam, The Netherlands; 3 Brain Research Center, Amsterdam, The Netherlands

**Keywords:** Neurofilament light protein, Serum, Biomarkers, Dementia, Alzheimer’s disease, Subjective Cognitive Decline, Clinical implementation, Diagnostic work-up

## Abstract

**Background:**

Neurofilament light in serum (sNfL) is a biomarker for axonal damage with elevated levels in many neurological disorders, including neurodegenerative dementias. Since within-group variation of sNfL is large and concentrations increase with aging, sNfL’s clinical use in memory clinic practice remains to be established. The objective of the current study was to evaluate the clinical use of serum neurofilament light (sNfL), a cross-disease biomarker for axonal damage, in a tertiary memory clinic cohort.

**Methods:**

Six neurologists completed questionnaires regarding the usefulness of sNfL (*n* = 5–42 questionnaires/neurologist). Patients that visited the Alzheimer Center Amsterdam for the first time between May and October 2019 (*n* = 109) were prospectively included in this single-center implementation study. SNfL levels were analyzed on Simoa and reported together with normal values in relation to age, as part of routine diagnostic work-up and in addition to cerebrospinal fluid (CSF) biomarker analysis.

**Results:**

SNfL was perceived as useful in 53% (*n* = 58) of the cases. SNfL was more often perceived as useful in patients < 62 years (29/48, 60%, *p* = 0.05) and males (41/65, 63%, *p* < 0.01). Availability of CSF biomarker results at time of result discussion had no influence. We observed non-significant trends for increased perceived usefulness of sNfL for patients with the diagnosis subjective cognitive decline (64%), psychiatric disorder (71%), or uncertain diagnosis (67%). SNfL was mostly helpful to neurologists in confirming or excluding neurodegeneration. Whether sNfL was regarded as useful strongly depended on which neurologist filled out the questionnaire (ranging from 0 to 73% of useful cases/neurologist).

**Discussion:**

Regardless of the availability of CSF biomarker results, sNfL was perceived as a useful tool in more than half of the evaluated cases in a tertiary memory clinic practice. Based on our results, we recommend the analysis of the biomarker sNfL to confirm or exclude neurodegeneration in patients below 62 years old and in males.

**Supplementary Information:**

The online version contains supplementary material available at 10.1186/s13195-021-00841-4.

## Background

Neurofilament light (NfL) is a major protein of the axonal cytoskeleton and an accurate blood biomarker for axonal damage across the spectrum of neurological diseases [1]. Increased levels of NfL in cerebrospinal fluid have been reported in many neurodegenerative and neurological diseases with estimated fold-changes of 1–5 for the dementias in comparison to healthy controls [2, 3]. Recent technological advancements allow the ultrasensitive detection of NfL in blood [4], facilitating an exponential increase in studies across the field of neurology that identify blood NfL as powerful biomarker for diagnostic, prognostic, or disease monitoring purposes [5–9]. Also, for neurodegenerative dementias, including Alzheimer’s disease (AD) and frontotemporal dementia (FTD), higher levels of blood NfL are informative biomarkers to separate dementia from controls on a group level [10–18].

The positive correlation of NfL levels with increasing age [2, 16, 19] complicates the interpretation of NfL and has delayed the establishment of cut-off values for clinical practice. Also, NfL levels across the different types of neurodegenerative dementias are largely overlapping [2], questioning the clinical value of NfL on the individual patient level in memory clinic practice. Prospective studies are thus needed to evaluate the usefulness of blood NfL in the diagnostic work-up for dementia, similar to the evaluation studies that were performed for the classical AD biomarkers in cerebrospinal fluid (CSF) [20, 21].

We here aimed to evaluate the clinical use of serum NfL (sNfL) in a prospective study performed in a tertiary memory clinic setting. Patients visiting the memory clinic were sampled for NfL analysis and neurologists per patient indicated how they appreciated the sNfL result. We analyzed the frequency of NfL results perceived as useful and studied whether patient characteristics, i.e., age, sex, and diagnosis, were associated with an increased percentage of perceived usefulness of sNfL.

## Methods

### Patients

We prospectively and consecutively included patients that had their first visit at the memory clinic of the Alzheimer Center Amsterdam between May and October 2019. The Alzheimer Center Amsterdam is a tertiary center, which means that patients are referred for analysis of their cognitive complaints by their general practitioner or via their local specialist physician, in case of second or third opinions, as is customary in the Netherlands [22]. At their first visit, all patients received a standardized and multidisciplinary work-up, including medical and neurological examination including history taking and cognitive examination by a neurologist, assessment of vital functions, informant based history, neuropsychological investigation, brain magnetic resonance imaging (MRI), electroencephalogram (EEG), standard laboratory work, and lumbar puncture for collection of cerebrospinal fluid (CSF). Clinical diagnosis was made by consensus in a multidisciplinary meeting. Subjects were diagnosed with subjective cognitive decline (SCD; *n* = 28) when cognitive complaints were present but criteria for mild cognitive impairment (MCI), dementia, or any other neurological or psychiatric disorders were not met and all other examinations were normal [23]. Subjects were diagnosed with MCI (*n* = 5), of which *n* = 3 due to AD and *n* = 2 clinically defined without underlying neurodegenerative disorders, or dementia due to Alzheimer’s disease (AD; *n* = 29), according to the established National Institute on Aging - Alzheimer’s Association diagnostic guidelines [24, 25]. Subjects were diagnosed with other types of dementia according to clinical guidelines for frontotemporal dementia (FTD; *n* = 3) [26, 27], dementia with Lewy bodies (DLB; *n* = 3) [28], primary progressive aphasia (PPA; *n* = 2) [29], progressive supranuclear palsy (PSP; *n* = 2) [30, 31], or vascular dementia (VaD; *n* = 1) [32]. Patients were diagnosed with psychiatric disorders and referred to a psychiatrist for further examination when signs of neurological or neurodegenerative diseases could not be objectified (*n* = 17). Some patients were diagnosed with other neurological diseases (*n* = 11), due to drug abuse (*n* = 2), Parkinson’s disease (*n* = 1), cerebral amyloid angiopathy (*n* = 1), temporal epilepsy (*n* = 1), recent subdural hematoma in combination with corticobasal syndrome (*n* = 1), suspected hydrocephalus (*n* = 2) in combination with AD (*n* = 1) or vascular damage or psychiatric disorders (*n* = 1), suspected functional disorder after cerebrovascular accident (*n* = 1), or unknown cause (*n* = 2). For some patients the diagnosis could not be established after the first visit and was therefore postponed (*n* = 8), of which *n* = 6 had a syndrome diagnosis of MCI or dementia, *n* = 1 had a suspected functional disorder, and for *n* = 1, a severe language barrier impeded further testing. Two weeks after the first visit, the neurologist discussed the diagnostic outcomes with the patient. All patients included in this study gave written informed consent for the storage of their clinical data for research purposes as part of the Amsterdam Dementia Cohort (ADC) and the ADC was approved by the local ethics committee [22, 33].

### Biomarker analysis

Blood and CSF samples were collected during routine diagnostic investigations and collected and processed at room temperature within 2 h (Alzheimer Center and Clinical Chemistry, Amsterdam UMC location VUmc, the Netherlands).

CSF biomarkers amyloid-beta(1-42) (aβ1-42), phosphorylated Tau(P181) (pTau), and total Tau (tTau) were analyzed using the Elecsys assays (Roche Diagnostics GmbH, Mannheim, Germany) as previously described [34, 35]. Cut-offs for biomarker abnormality were determined at < 1000 pg/mL for aβ1-42, > 19 pg/mL for pTau and > 235 pg/mL for tTau [34, 35].

Serum was stored at − 20 °C for 1–7 days until the weekly routine analysis of NfL. Serum was thawed at room temperature and centrifuged for 10 minutes at 10,000*g*. Serum was diluted 1:4 and analyzed on Simoa HD-1 (Quanterix, Lexington, MA) using the commercially available NF-Light kit (Quanterix, Lexington, MA) according to manufacturer’s instructions. The intra-assay coefficient of variation (CV; mean ± SD) for NfL was 3.5 ± 0.8 %, calculated as the average of the duplo CV of samples per run, averaged over all runs (*n* = 25). The inter-assay CV percentage for NfL was calculated for three human quality control (QC) serum samples over all the runs (*n* = 25): 7.9 % for QC high, 10.0% for QC medium, and 7.2% for QC low.

### Questionnaires

Two weeks after the examination day, patients returned to the clinic to discuss their diagnostic result with their neurologist. After this visit, neurologists filled out a questionnaire regarding the use of sNfL. The questionnaire inquired after the suspected diagnosis, including the differential diagnosis or other relevant notes, and asked how the neurologist used the NfL result. The answer to the latter question was one of the following options: “No, not used for diagnosis”; “Yes, confirmation of diagnosis”; “Yes, exclude diagnosis, namely…”; “Yes, differentiate between different forms of dementia”; “Yes, to comfort the patient”; “Yes, prognostic”; “Yes, for trial selection”; “Yes, different, namely….” The backside of the form included a graph and table with the sNfL reference range of healthy controls in relation to age (Additional file 1, Figure e-1 and Table e-1).

Background information regarding the years of experience as neurologist, time spent on research, frequency of performing result consultations, and previous knowledge and experience with sNfL were requested from the participating neurologists.

### Data analysis

To define the usefulness of sNfL, the answer to the question how the neurologist used the NfL result was dichotimized as “yes” (“Yes, confirmation of diagnosis”; “Yes, exclude diagnosis, namely…”; “Yes, differentiate between different forms of dementia”; “Yes, to comfort the patient”; “Yes, prognostic”; “Yes, for trial selection”; “Yes, different, namely…”; and “no” (“No, not used for diagnosis”). Diagnoses that had less than five patients per group, i.e., FTD (*n* = 3), DLB (*n* = 3), PPA (*n* = 2), PSP (*n* = 2), and VAD (*n* = 1), were grouped together as “other dementia (OD)” for further analyses. The variable “doubt regarding diagnosis” was constructed based on whether the neurologist indicated one diagnosis (doubt: “no”) or more than one diagnosis or an additional comment on the questionnaire (doubt: “yes”). To study the reasons underlying the use of NfL, patients with a diagnosis of MCI, AD, or OD were grouped as “neurodegenerative diseases,” patients with SCD or psychiatric disorder as “no neurodegenerative diseases,” and patients with other neurology or a postponed diagnosis as “other.”

Demographics were stratified for neurologist (Additional file 1, Table e-2), and variables were tested group-wise using Fisher exact test for categorical variables, one-way analysis of variance (ANOVA) for normally distributed continuous variables, and Kruskal Wallis test for non-normally distributed continuous variables. Logistic regression analysis was performed to assess the effect of age on the perceived usefulness of sNfL with neurologist as covariate. For the categorical predictors, i.e., age categories or dichotomized, sex, availability of CSF biomarker results, doubt regarding diagnosis or diagnosis, mixed models were applied with neurologist as random intercept. Linear regression analysis was performed to study the effect of age on log-transformed sNfL concentrations. A linear mixed model was applied to assess the differences in log-transformed sNfL concentrations across the diagnostic groups, correcting for age. Post-hoc pairwise comparisons were performed using the “emmeans” package with Tukey *p* value adjustment for multiple testing. Non-parametric Mann-Whitney *U* tests were performed to compare sNfL concentrations between CSF amyloid status (CSF aβ1-42), CSF tau status (CSF pTau), and CSF neurodegeneration status (CSF tTau). Statistical analyses were performed in R version 4.0.2 [36], and *p* values below 0.05 were considered significant.

## Results

### Questionnaire responses

In total, 109 questionnaires were completed by six neurologists (Table 1). In more than half of the questionnaires (*n* = 58, 53%), the neurologist perceived the sNfL result as useful. Two neurologists (A and E) together filled out the majority of all questionnaires (*n* = 82, 75%) and perceived sNfL as useful in 71% of the questionnaires. The other four neurologists each completed 5-10 questionnaires and never perceived sNfL as useful. There was variation across the neurologists’ backgrounds: the number of years working as a neurologist at time of the study ranged from 1 to 30 years; time spent on research varied between 20 and 60%; the frequency of performing result consultations varied from weekly to once every 2 weeks to incidentally. Four neurologists (A, C, D, and E) had prior knowledge on sNfL and two had no or very little, while only one neurologist (C) had used sNfL as biomarker prior to the current study.

**Table 1:** Frequencies of questionnaire responses across six neurologists of the Alzheimer Center Amsterdam and details on neurologists’ background. Abbreviation: sNfL, serum Neurofilament Light.

	Total	Neurologist					
		A	B	C	D	E	F
Number of completed questionnaires (%)	109	40 (37)	6 (6)	6 (6)	10 (9)	42 (39)	5 (5)
sNfL useful?							
Yes, *n* (%)	58 (53)	29 (73)	0 (0)	0 (0)	0 (0)	29 (69)	0 (0)
No, *n* (%)	51 (47)	11 (27)	6 (100)	6 (100)	10 (100)	13 (31)	5 (100)
Years as neurologist		4	3	14	18	30	1
Time spent on research (%)		40%	60%	20%	25%	40%	50%
Frequency of performing results consultations		1x/week	1x/2 weeks	1x/week(on average)	Incidental	1x/week	1x/2 weeks
Had prior knowledge of sNfL		Yes	No	Yes	Yes	Yes	Very little
Had prior experience with sNfL as biomarker		No	No	No	Yes	No	No

### Cohort characteristics

For 109 patients, a questionnaire was completed (Table 2). The average age of the patients was 63 ± 9 years old, 40% was female, and the median mini-mental state examination (MMSE) score was 26. For 69% of the patients, biomarker results for CSF aβ_1-42_, pTau and tTau were available. For 17% of the patients, neurologists indicated to have a doubt regarding the diagnosis. Patients were mainly diagnosed with AD, SCD, or psychiatric diagnoses. The demographics of this study were comparable to those of the ADC [33], indicating that a representative sample of the tertiary memory clinic population was included in the current study. To examine potential bias in the type of patients seen by the different neurologists, the cohort was additionally stratified by neurologist (see Additional file 1 Table e-2). Other than neurologists A and E seeing all the FTD and PPA patients (*n* = 5 in total), the distributions of the patient characteristics among neurologists were in proportion to the total cohort and the number of completed questionnaires per neurologist.

**Table 2:** Patient demographics of the cohort that was prospectively studied for the neurologist’s perceived usefulness of biomarker sNfL

	Total
*n*	109
Age, mean (SD)	63 (9)
Sex = f, *n* (%)	44 (40)
MMSE, median [IQR]	26 [23, 28]
CSF biomarkers available = Y (%)	76 (69)
Doubt diagnosis = yes (%)	18 (17)
Diagnosis, *n* (%)	
Subjective cognitive decline (SCD)	28 (26)
Mild cognitive impairment (MCI), of which 3 due to AD, 2 clinically defined	5 (5)
Dementia due to AD	29 (27)
Other dementia (OD)	11 (10)
Other neurology (ON)	11 (10)
Psychiatric disorder	17 (16)
Postponed	8 (7)
aβ1-42, pg/mL (median [IQR])	889 [559, 1446]
pTau, pg/mL (median [IQR])	16 [11, 26]
tTAU, pg/mL (median [IQR])	200 [135, 290]
sNfL, pg/mL (median [IQR])	14 [10, 19]

Variables are represented as number (percentage) or as median [interquartile range] concentration in pg/mL

Abbreviations: amyloid-beta1-42: aβ1-42; CSF: cerebrospinal fluid; MMSE: Mini Mental State Examination; pTau: phosphorylated Tau; sNfL: serum Neurofilament Light; tTau: total Tau

### Serum NfL is more often perceived as useful in younger patients

**Fig. 1:**
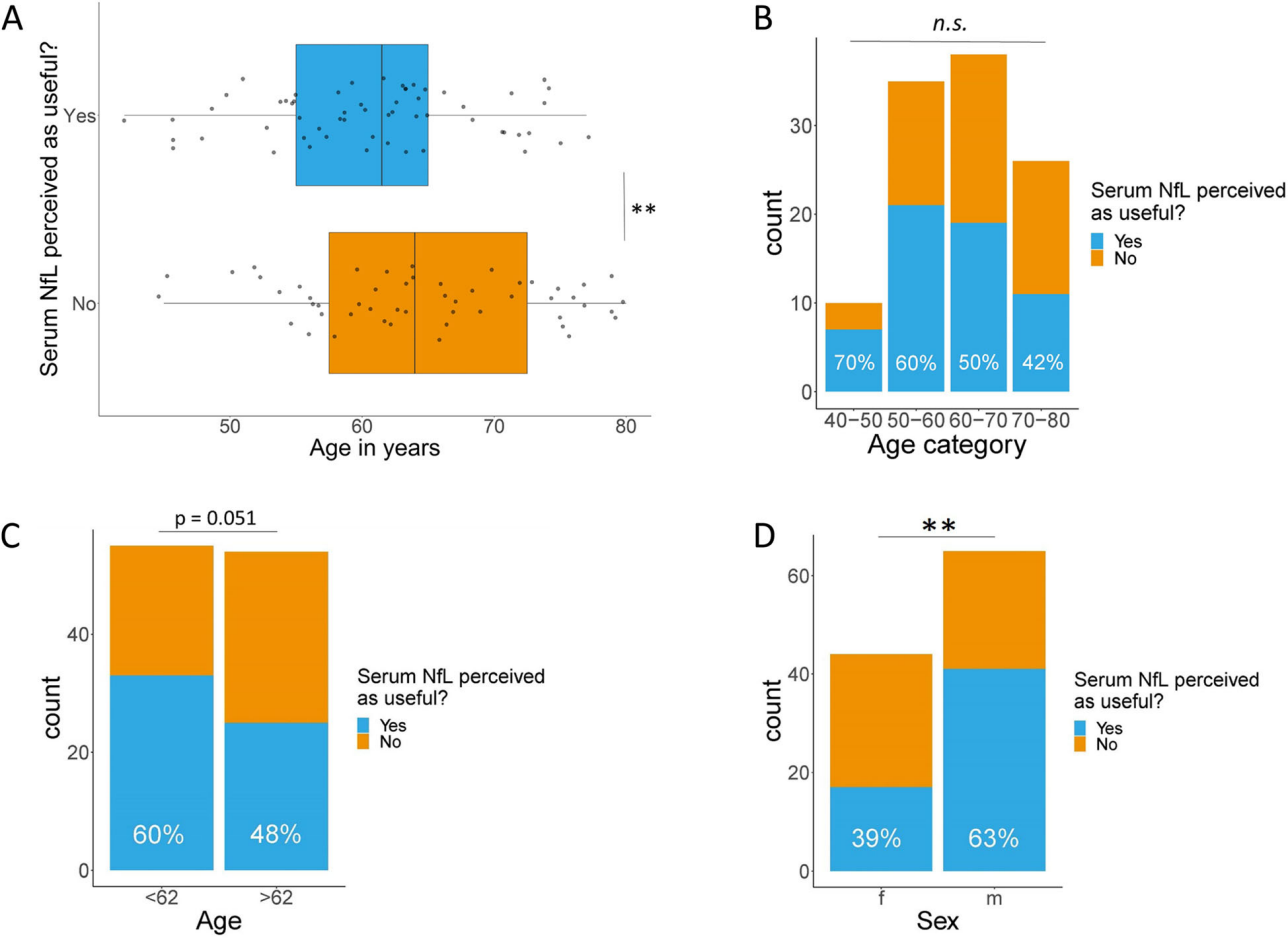
Perceived usefulness of sNfL in relation to age and sex. **a** Age as continuous variable. **b** Age as categorical variable. **c** Age dichotomized at the median. **d** Sex. Abbreviations: f, female; m, male; sNfL, serum neurofilament light; N.s., not significant, *p value < 0.05; **p value < 0.01, ***p value < 0.001

Age was a significant predictor of the perceived usefulness of sNfL with a beta estimate of -0.0975 ± 0.0331 (*p* < 0.005). Thus, patients in which sNfL was perceived as useful were on group level younger than patients in which sNfL was not perceived as useful (Fig. 1a). Zooming in on age categories, the perceived usefulness of sNfL decreased with every decade of older age: in 70% of subjects aged 40–50 years, sNfL was perceived as useful; in subjects aged 50–60 years, this was 60%; in subjects aged 60–70 years, this was 50%, compared to 42% in those aged 70–80 years, but these differences were not statistically significant (Fig. 1b). When dichotomizing age at the median (62 years), sNfL was perceived as useful in 60% of the youngest half of the cohort and in 48% of the oldest half of the cohort, which was borderline significant (*p* = 0.051, Fig. 1c).

### Serum NfL is more often perceived as useful in male patients

Sex was a significant predictor of the perceived usefulness of sNfL with a beta estimate of 1.58 ± 0.52 (*p* < 0.005). Patients in which sNfL was perceived as useful were more often male than female (Fig. 1d). After adjusting for age, the effect of sex on the perceived usefulness of sNfL was even stronger, with a beta estimate of 1.98 ± 0.62 (*p* < 0.005). Age distribution was similar between males and females with 60% males in the youngest group (< 62 years) and 59% males in the oldest group (> 62 year).

### Perceived usefulness of sNfL does not depend on CSF biomarker availability

**Fig. 2:**
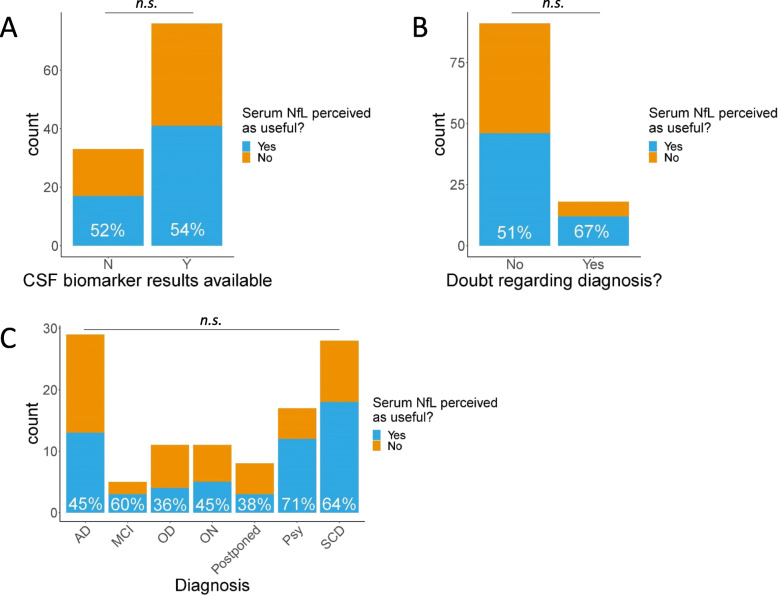
Perceived usefulness of sNfL in relation to availability of CSF biomarker results (**a**); doubt regarding the diagnosis (**b**); and diagnosis (**c**). Abbreviations: AD, Alzheimer’s disease; MCI, mild cognitive impairment; OD, other dementia; ON, other neurology; Psy, psychiatric disorder; N, no; Y, yes; N.s., not significant; *p value < 0.05; **p value < 0.01, ***p value < 0.001

In the 33 (30%) patients of which no CSF biomarker result was available, sNfL was perceived as useful in 52%, which was a similar proportion as in patients with a CSF biomarker result (54%; Fig. 2a).

### Perceived usefulness of sNfL in relation to doubt regarding the diagnosis

In 18 (17%) patients, the neurologist expressed a doubt regarding the diagnosis. In 67% of these patients, sNfL was perceived as useful, while for the patients without uncertainty regarding their diagnosis, 51% of the sNfL results were perceived as useful (Fig. 2b). This difference was not statistically significant.

### Perceived usefulness of serum NfL in relation to diagnosis

Serum NfL was more often perceived as useful in patients with a diagnosis of psychiatric disorder (71%) or SCD (64%) in comparison to a diagnosis of AD (45%); however, these differences did not reach statistical significance, also not when corrected for age and gender (Fig. 2c). The psychiatric disorder and SCD groups had a male predominance, with 12 out of 17 (71%) males in the psychiatric disorder group and 19 out of 28 (68%) in the SCD group, compared to 15 out of 29 (48%) in the AD group, which may underlie the trend in differences in perceived usefulness across diagnoses.

### Serum NfL was perceived as useful mainly to confirm or exclude neurodegeneration

Next, we zoomed in on the cases in which sNfL was perceived as useful and listed the reasons of sNfL’s contribution as indicated by the neurologist (Table 3). We grouped the different diagnoses into neurodegenerative diagnosis (*n* = 20, 34%), no neurodegenerative diagnosis (*n* = 30, 52%), and other neurology or postponed diagnosis (*n* = 8, 14%). Reasons for perceived usefulness of sNfL were often quite similar, e.g., “excluding neurodegeneration” and “confirm no neurodegeneration,” or with subtle differences, e.g., “confirm neurodegeneration” versus “support neurodegeneration.” Upon grouping reasons that point in the same direction, the summarized main reasons for perceived usefulness of sNfL were per group: confirming neurodegeneration in cases with neurodegeneration (75%), excluding neurodegeneration in cases without neurodegeneration (87%) and to either confirm or exclude neurodegeneration in 63% of the other neurology or postponed cases. We also observed that in a few patients, sNfL was perceived as useful for follow-up, as indicator of vessel damage, or was explained by a recent heart attack.

**Table 3:** Reasons for neurologists on how they used the sNfL result in case they perceived it as useful

Neurodegenerative diagnosis (***n*** = 20)		No neurodegenerative diagnosis (***n*** = 30)		Other (***n*** = 8)	
Confirm AD	*N* = 10	Exclude neurodegeneration	*N* = 8	Confirm neurological disorder	*N* = 3
Confirm neurodegeneration, e.g., MCI, FTD, other	*N* = 5	Confirm SCD	*N* = 6	Exclude AD	*N* = 1
Support AD	*N* = 2	Confirm no neurodegeneration	*N* = 4	Exclude neurodegeneration	*N* = 1
Support neurodegeneration	*N* = 1	To reassure patient	*N* = 4	For follow-up	*N* = 1
Exclude FTD, confirm AD	*N* = 1	Exclude FTD	*N* = 2	Due to recent heart attack	*N* = 1
For follow-up	*N* = 1	Less support for FTD	*N* = 1	Other	*N* = 1
		Exclude AD	*N* = 1		
		Exclude CTE	*N* = 1		
		Support SCD	*N* = 1		
		For patient management	*N* = 1		
		Indication for vessel damage	*N* = 1		

“Neurodegeneration” included subjects diagnosed with MCI, AD, or OD; “no neurodegeneration” included subjects with SCD or psychiatric disorder; “other” included subjects with other neurology or a postponed diagnosis. Abbreviations: *AD* Alzheimer’s disease, *MCI* mild cognitive impairment

### Serum NfL concentrations increase with age, differ across diagnoses, and are elevated in CSF biomarker positive cases

**Fig. 3:**
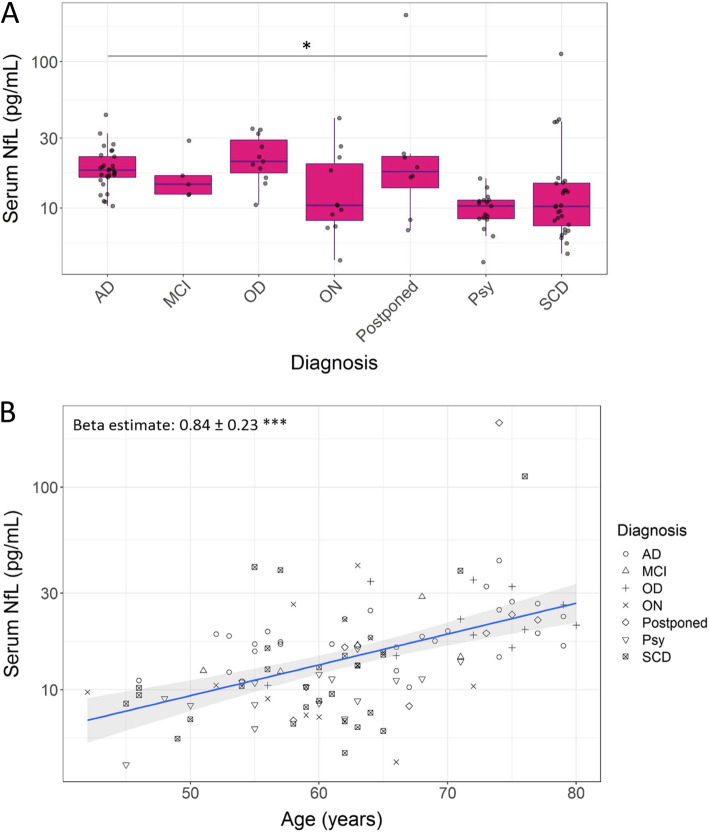
Concentrations of sNfL across different diagnoses (**a**) and in relation to age (**b**). Abbreviations: AD, Alzheimer’s disease; MCI, mild cognitive impairment; OD, other dementia; ON, other neurology; Psy, psychiatric disorder; SCD, subjective cognitive decline. *p value < 0.05; **p value < 0.01, ***p value < 0.001

The concentrations of sNfL increased with age in our total cohort (Fig. 3b). The increase of sNfL (± standard error) was on average 0.84 ± 0.23 pg/mL per year (*p* < 0.001). We also found trends for differences in the average sNfL concentrations across the diagnostic groups after correcting for age (Fig. 3a). The median [interquartile range (IQR)] sNfL concentrations per diagnostic group were 18 [6.2] pg/mL for AD, 15 [4.2] pg/mL for MCI, 21 [12] pg/mL for OD, 10 [12] for ON, 18 [8.2] pg/mL for postponed, 10 [2.9] pg/mL for psychiatric disorder, and 10 [7.2] pg/mL for SCD. The only significant difference in pairwise post-hoc comparisons after correction for multiple testing was reported between the AD and psychiatric disorder groups (*p* < 0.05). SNfL levels were elevated in positive versus negative CSF biomarker profiles (median [IQR]): 16.2 [8.1] pg/mL versus 11.1 [6.7] pg/mL for CSF amyloid status (*p* < 0.05); 16.6 [5.1] pg/mL versus 10.5 [7.5] pg/mL for CSF tau status (*p* < 0.001); and 16.6 [5.1] pg/mL versus 10.5 [7.5] pg/mL for CSF neurodegeneration status (*p* < 0.001).

## Discussion

Literature supporting blood NfL as biomarker for neurodegenerative disorders is expanding, but prospective studies to support the clinical use of sNfL for individual patients are lacking. Our study prospectively and systematically monitored the added value of sNfL for individual patients in tertiary memory clinic setting from the neurologist’s perspective. We found that in more than half (53%) of the patients visiting the memory clinic, the treating neurologist evaluated the sNfL result as useful, mainly to detect neurodegeneration. SNfL results were evaluated as part of the diagnostic workup at patients’ first visit. We identified patient characteristics that increased the perceived usefulness of sNfL, such as younger age and male sex, and additionally observed a bias in perceived usefulness across neurologists.

Our main result showed that sNfL was perceived as useful by neurologists mostly in younger patients (< 62 years, 60%) and in male patients (63%). These characteristics were independent predictors of sNfL’s perceived usefulness, meaning that gender and age were not related; males were not younger than females in our cohort. We did not find a significant effect of diagnosis on the perceived usefulness of sNfL, although sNfL was more often perceived as useful in the SCD and psychiatric disorder group (64 and 71%). Interestingly, younger age and male gender were predominant characteristics in the SCD and psychiatric disorder groups, which may have biased the effect of suspected diagnosis on the perceived usefulness of sNfL. The effect of diagnosis, in particular that of SCD or psychiatric disorder, might be underestimated in our study due to the overrepresentation of young male patients in these groups. A typical clinical picture of neurodegeneration versus non-neurodegeneration is that of a young, male patient that enters the memory clinic with a suspected diagnosis of SCD or psychiatric disorder versus neurodegeneration, for example in phenocopy FTD syndrome [37, 38]. Our results support sNfL as helpful biomarker for such clinical questions in particular [12, 17, 18].

We anticipated that sNfL would be more often interpreted as useful in subjects without CSF biomarker results, due to the paucity of add-on objective tests to sustain the diagnosis, but surprisingly, the availability of CSF biomarker results had no influence on the perceived usefulness of sNfL. This indicates that sNfL contributes additional information on top of the information provided by the CSF biomarker results. The CSF biomarkers reflect amyloid plaque pathology (aβ1-42), tau tangle pathology (pTau), and neurodegeneration (tTau), pathologies specific to AD, while sNfL reflects axonal degeneration which occurs in various neurological disorders and is thus also elevated in various disorders. In dementias other than AD, e.g., FTD or PSP, CSF biomarkers are often in the normal range and sNfL will be useful as indicator for an ongoing neurodegenerative process. NfL thus represents an additional pathology, axonal degeneration, that is a useful biomarker of neurodegenerative pathology.

In subjects for which neurologists expressed doubt regarding the diagnosis, sNfL was perceived as useful in 67% of the cases, compared to in 51% of the subjects in which no doubt regarding diagnosis was expressed. This difference was not statistically significant, which could be a power issue, as the first group comprised less than 20% of the total cohort. Replicate studies should clarify whether sNfL has added value as biomarker in memory clinic patients with indecisive diagnoses.

The main use of sNfL reported by neurologists was either to confirm or to exclude neurodegeneration. In 75% of the subjects with an indication for neurodegenerative disease in which sNfL was perceived as useful, the reason was confirming neurodegeneration in general, or specified as AD, FTD, MCI, or other. Other reasons were supporting neurodegeneration, differentiating between AD and FTD, or for follow-up. In the subjects without an indication for neurodegeneration in which sNfL was perceived as useful, the reason was in 87% summarized as excluding neurodegeneration. In the “other“ diagnostic group, in subjects for which sNfL was perceived as useful, in 63%, the reason was to confirm or exclude neurodegeneration, depending on the suspected diagnosis. Interestingly, for two patients, neurologists indicated that the sNfL result was useful because they related it to a recent heart attack or interpreted it as an indication for vessel damage. Since NfL is not specific to neurodegenerative diseases, it is important to consider other underlying diseases as cause of increased NfL levels, especially in the elderly that often suffer from co-pathologies, such as cardiovascular problems [39].

Regarding the measured levels, sNfL levels were lowest in the psychiatric disorder and SCD groups and highest in the AD, OD, and postponed diagnosis groups, with a statistically significant difference after age correction between the psychiatric disorder and AD groups only. These findings are in line with previous literature [2, 10, 15, 16]. We observed two outliers with sNfL values > 100 pg/mL: one subject had a postponed diagnosis with suspicion of vascular dementia; the other had a diagnosis of SCD with underlying cerebral amyloid angiopathy. Literature supports a role for sNfL in cardiovascular diseases [40–42]. Multiple studies including ours showed an increase of NfL in blood with age and an increase in the variation of NfL levels with more outliers in the higher concentration range starting from the age of 60 [19, 43, 44]. These outliers presumably reflect underlying pathologies in the CNS, e.g., neurodegeneration or hemorrhage, in the preclinical stage [19]. Such high variation in normal NfL ranges in older age might explain the added value of sNfL in the younger population in particular that we observed in the current study.

## Limitations

This study also faced some limitations. First, there was a strong bias by neurologist in the number of completed questionnaires as well as in the evaluation of sNfL as useful tool. Although we corrected for neurologist in all statistical analyses, personal experiences of neurologists might have affected the outcomes of our study. We closely examined neurologists’ background and the type of patients they have seen in this study and could not identify direct explanations. A combination of factors likely explains the bias, i.e., number of cases evaluated by the neurologist, his/her experience level with sNfL, and the type of patient in front of him/her. Second, our cohort had low power to assess the effects of the diagnostic group on the perceived usefulness of sNfL, for which independent replication studies will be needed. Third, this study was performed at one memory clinic that is specialized in early onset dementia. Results should therefore first be replicated in an independent cohort before these may be generalized to other memory clinic populations.

## Conclusions

In conclusion, we here objectified the usefulness of sNfL in a prospective setting by monitoring the neurologists’ opinion on the added value of NfL for the individual patient. Since NfL is not specific for a specific brain disease, its practical use in the memory clinic was not a priori defined despite convincing retrospective cohort studies in literature. Our study revealed that in a memory clinic population, sNfL is deemed useful, especially in younger and in male patients and regardless of the availability of CSF biomarker results. Also, a bias between neurologists based on their background, type of patients seen, and personal experience influenced the perceived usefulness of sNfL. Our results provide first evidence on the clinical context of use of sNfL in memory clinic practice and need replication in other centers to form a basis for future clinical guidelines.

## Supplementary Information

**Additional file 1.** Serum NfL reference values reported to neurologists together with sNfL results (Figure e-1 and Table e-1) and patient demographics stratified per neurologist (Table e-2).

## Data Availability

The datasets used and/or analyzed during the current study are available from the corresponding author on reasonable request.

## Abbreviations

aβ1-42: Amyloid-beta(1-42)
AD: Alzheimer’s disease
ADC: Amsterdam Dementia Cohort
CNS: Central nervous system
CSF: Cerebrospinal fluid
cv: Coefficient of variation
DLB: Dementia with Lewy bodies
EEG: Electroencephalogram
FTD: Frontotemporal dementia
IQR: Interquartile range
MCI: Mild cognitive impairment
MRI: Magnetic resonance imaging
PPA: Primary progressive aphasia
PSP: Progressive supranuclear palsy
pTau: Phosphorylated Tau(P181)
Psy: Psychiatric disorder
QC: Quality control
SCD: Subjective cognitive decline
tTau: Total Tau
sNfL: Serum neurofilament light
VaD: Vascular dementia
