# Supplementary material for: A neurologist’s perspective on serum neurofilament light in the memory clinic: a prospective implementation study
Source: Alzheimers Res Ther. 2021 May 18;13:101. doi: 10.1186/s13195-021-00841-4 (PMC8132439; doi:10.1186/s13195-021-00841-4)
Supplement: Supplementary file 1 — Additional file 1. Serum NfL reference values reported to neurologists together with sNfL results (Figure e-1 and Table e-1) and patient demographics stratified per neurologist (Table e-2). [file 13195_2021_841_MOESM1_ESM.docx]

**Additional file 1**

**A neurologist’s perspective on serum Neurofilament Light in the memory clinic: a prospective implementation study**

E. A. J. Willemse^1^, P. Scheltens^2^, C. E. Teunissen^1^, E. G. B. Vijverberg^2,3^

^1^ Neurochemistry laboratory, Department of Clinical Chemistry, Amsterdam Neuroscience, Amsterdam University Medical Center, Vrije Universiteit, De Boelelaan 1117, Amsterdam, The Netherlands

^2^ Alzheimer Center, Department of Neurology, Amsterdam Neuroscience, Amsterdam University Medical Center, Vrije Universiteit, De Boelelaan 1117, Amsterdam, The Netherlands

^3^ Brain Research Center, Amsterdam, The Netherlands

Figure e-1 shows the 95% prediction interval for NfL serum concentrations (on a logarithmic scale) in healthy subjects. These data are based on a reference group of 343 healthy persons aged 19-98 years. Table e-1 shows the 95% prediction intervals per age. Data were acquired at the Neurochemistry laboratory, department of Clinical Chemistry, Amsterdam University Medical Center location VUmc, Amsterdam, The Netherlands.

| **Figure e-1: Serum NfL results from healthy controls in relation to age.** The blue line represents the reference concentration including the 95% confidence interval for the present cohort. The red dotted lines represent the 95% prediction interval which is the expected result for a new measurement. |
| --- |
| 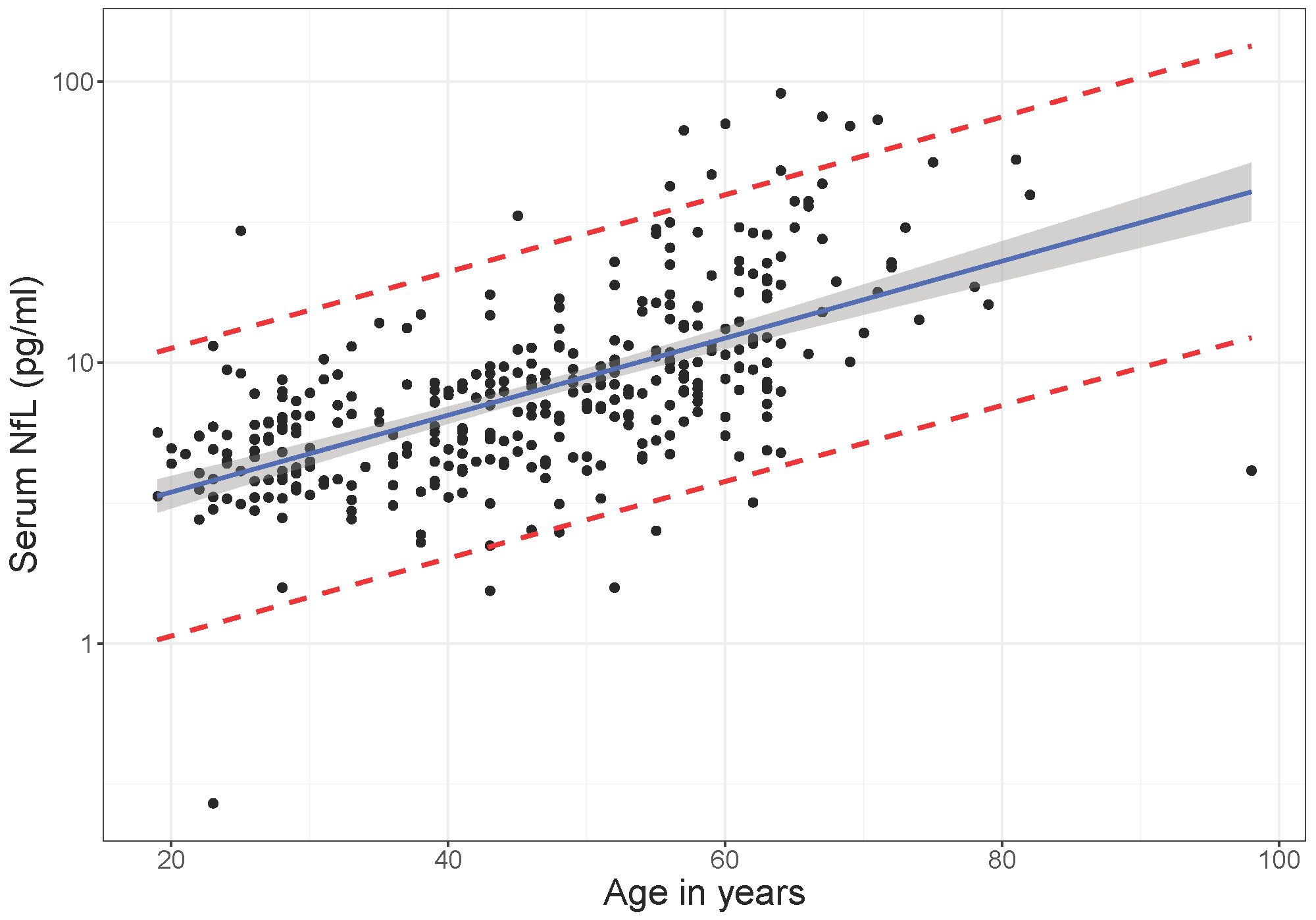 |

| **Table e-1: Serum NfL (pg/ml) reference ranges per age** |
| --- |
| \| Age in years \| NfL concentration (pg/ml)  [95% confidence interval]  (= blue line and shade) \| 95% prediction interval  (= red lines) \| \| --- \| --- \| --- \| \| 20 \| 3 [3 – 4] pg/ml \| 1 – 11 pg/ml \| \| 25 \| 4 [4 – 5] pg/ml \| 1 – 13 pg/ml \| \| 30 \| 5 [4 – 5] pg/ml \| 1 – 15 pg/ml \| \| 35 \| 6 [5 – 6] pg/ml \| 2 – 18 pg/ml \| \| 40 \| 7 [6 – 7] pg/ml \| 2 – 21 pg/ml \| \| 45 \| 8 [7 – 8] pg/ml \| 2 – 25 pg/ml \| \| 50 \| 9 [8 – 10] pg/ml \| 3 – 29 pg/ml \| \| 55 \| 10 [10 – 11] pg/ml \| 3 – 34 pg/ml \| \| 60 \| 12 [11 – 13] pg/ml \| 4 – 40 pg/ml \| \| 65 \| 14 [13 – 16] pg/ml \| 4 – 46 pg/ml \| \| 70 \| 17 [15 – 19] pg/ml \| 5 – 54 pg/ml \| \| 75 \| 20 [17 – 23] pg/ml \| 6 – 64 pg/ml \| \| 80 \| 24 [20 – 28] pg/ml \| 7 – 77 pg/ml \| |

Table e-2. Patient demographics stratified for neurologist.

|  | **Overall** | Neurologist A | Neurologist B | Neurologist C | Neurologist D | Neurologist E | Neurologist F | p |
| --- | --- | --- | --- | --- | --- | --- | --- | --- |
| n | **109** | 40 | 6 | 6 | 10 | 42 | 5 |  |
| sNfL useful = Yes (%) | **58 (53)** | 29 (72.5) | 0 (0.0) | 0 (0.0) | 0 (0.0) | 29 (69.0) | 0 (0.0) | <0.001 |
| **Diagnosis, n (%)** |  |  |  |  |  |  |  |  |
| Dementia due to AD | **29 (26.6)** | 9 (22.5) | 3 (50.0) | 1 (16.7) | 2 (20.0) | 12 (28.6) | 2 (40.0) |  |
| Dementia with Lewy Bodies (DLB) | **3 (2.8)** | 1 (2.5) | 0 (0.0) | 0 (0.0) | 1 (10.0) | 1 (2.4) | 0 (0.0) |  |
| Frontotemporal dementia (FTD) | **3 (2.8)** | 1 (2.5) | 0 (0.0) | 0 (0.0) | 0 (0.0) | 2 (4.8) | 0 (0.0) |  |
| Mild cognitive impairment (MCI) | **5 (4.6)** | 1 (2.5) | 1 (16.7) | 0 (0.0) | 0 (0.0) | 3 (7.1) | 0 (0.0) |  |
| Other dementia (OD) | **2 (1.8)** | 0 (0.0) | 0 (0.0) | 0 (0.0) | 0 (0.0) | 2 (4.8) | 0 (0.0) |  |
| Other neurology (ON) | **11 (10.1)** | 2 (5.0) | 0 (0.0) | 2 (33.3) | 0 (0.0) | 7 (16.7) | 0 (0.0) |  |
| Postponed | **8 (7.3)** | 4 (10.0) | 1 (16.7) | 1 (16.7) | 1 (10.0) | 1 (2.4) | 0 (0.0) |  |
| Primary progressive Aphasia (PPA) | **2 (1.8)** | 0 (0.0) | 0 (0.0) | 0 (0.0) | 0 (0.0) | 2 (4.8) | 0 (0.0) |  |
| Psychiatric disorder | **17 (15.6)** | 7 (17.5) | 1 (16.7) | 2 (33.3) | 2 (20.0) | 5 (11.9) | 0 (0.0) |  |
| Subjective cognitive decline (SCD) | **28 (25.7)** | 14 (35.0) | 0 (0.0) | 0 (0.0) | 4 (40.0) | 7 (16.7) | 3 (60.0) |  |
| Vascular dementia (VaD) | **1 (0.9)** | 1 (2.5) | 0 (0.0) | 0 (0.0) | 0 (0.0) | 0 (0.0) | 0 (0.0) |  |
| **Grouped diagnosis, n (%)** |  |  |  |  |  |  |  |  |
| Dementia due to AD | **29 (26.6)** | 9 (22.5) | 3 (50.0) | 1 (16.7) | 2 (20.0) | 12 (28.6) | 2 (40.0) |  |
| Mild cognitive impairment (MCI) | **5 (4.6)** | 1 (2.5) | 1 (16.7) | 0 (0.0) | 0 (0.0) | 3 (7.1) | 0 (0.0) |  |
| Other dementia (OD) | **11 (10.1)** | 3 (7.5) | 0 (0.0) | 0 (0.0) | 1 (10.0) | 7 (16.7) | 0 (0.0) |  |
| Other neurology (ON) | **11 (10.1)** | 2 (5.0) | 0 (0.0) | 2 (33.3) | 0 (0.0) | 7 (16.7) | 0 (0.0) |  |
| Postponed | **8 (7.3)** | 4 (10.0) | 1 (16.7) | 1 (16.7) | 1 (10.0) | 1 (2.4) | 0 (0.0) |  |
| Psychiatric disorder | **17 (15.6)** | 7 (17.5) | 1 (16.7) | 2 (33.3) | 2 (20.0) | 5 (11.9) | 0 (0.0) |  |
| Subjective cognitive decline (SCD) | **28 (25.7)** | 14 (35.0) | 0 (0.0) | 0 (0.0) | 4 (40.0) | 7 (16.7) | 3 (60.0) |  |
| Doubt diagnosis = Yes (%) | **18 (16.5)** | 9 (22.5) | 1 (16.7) | 1 (16.7) | 1 (10.0) | 5 (11.9) | 1 (20.0) | 0.844 |
| Age, years (mean (SD)) | **63 (9)** | 64 (8) | 65 (7) | 60 (10) | 61 (12) | 61 (9) | 64 (5) | 0.621 |
| Sex = m, n (%) | **65 (59.6)** | 25 (62.5) | 3 (50.0) | 4 (66.7) | 7 (70.0) | 24 (57.1) | 2 (40.0) | 0.870 |
| MMSE, median [IQR] | **26 [23, 28]** | 26 [24, 27] | 27 [25, 28] | 25 [24, 26] | 27 [25, 29] | 26 [23, 28] | 25 [23, 28] | 0.927 |
| sNfL, pg/mL (median [IQR]) | **14 [10, 19]** | 13 [8, 18] | 17 [13, 23] | 9 [8, 11] | 14 [9, 21] | 16 [11, 22] | 13 [10, 16] | 0.179 |
| CSF biomarkers available = Y (%) | **76 (69.7)** | 34 (85.0) | 4 (66.7) | 5 (83.3) | 6 (60.0) | 25 (59.5) | 2 (40.0) | 0.056 |
| aβ1-42, pg/mL (median [IQR]) | **889 [559, 1446]** | 1017 [559, 1480] | 1065 [618, 1498] | 1506 [406, 1700] | 882 [725, 1247] | 787 [630, 959] | 768 [613, 923] | 0.791 |
| pTau, pg/mL (median [IQR]) | **16 [11, 26]** | 15 [12, 23] | 25 [20, 28] | 15 [11, 16] | 9 [8, 13] | 25 [12, 32] | 32 [31, 33] | 0.013 |
| tTAU, pg/mL (median [IQR]) | **200 [135, 290]** | 193 [135, 252] | 325 [270, 335] | 178 [128, 190] | 104 [90, 148] | 261 [156, 318] | 358 [337, 379] | 0.014 |

Variables are represented as number (percentage) or as median [interquartile range, IQR] concentration in pg/mL. Pairwise comparisons were performed using the Fisher exact test for dichotomous variables and analysis of variance or Kruskall Wallis for continuous variables. Abbreviations: amyloid-beta1-42: aβ1-42; CSF: cerebrospinal fluid; MMSE: Mini Mental State Examination; NA: not applicable; pTau: phosphorylated Tau; sNfL: serum Neurofilament Light; tTau: total Tau.
